# 2-(4-Meth­oxy-1*H*-indol-3-yl)acetonitrile

**DOI:** 10.1107/S1600536811053360

**Published:** 2011-12-17

**Authors:** Yong-Hong Lu, Mei-Ling Pan, Yang-Hui Luo

**Affiliations:** a Ordered Matter Science Research Center, College of Chemistry and Chemical Engineering, Southeast University, Nanjing 210096, People’s Republic of China

## Abstract

In the title compound, C_11_H_10_N_2_O, the cyanide group is twisted away from the indole-ring plane [C_cy_—C_me_—C_ar_—C_ar_ = 70.7 (2)°; cy = cyanide, me = methyl­ene, ar = aromatic], whereas the meth­oxy C atom is almost coplanar with the ring system [displacement = 0.014 (5) Å]. In the crystal, N—H⋯N hydrogen bonds link the mol­ecules into *C*(7) chains propagating in [100].

## Related literature

For a related structure, see: Ge *et al.* (2012 ▶).
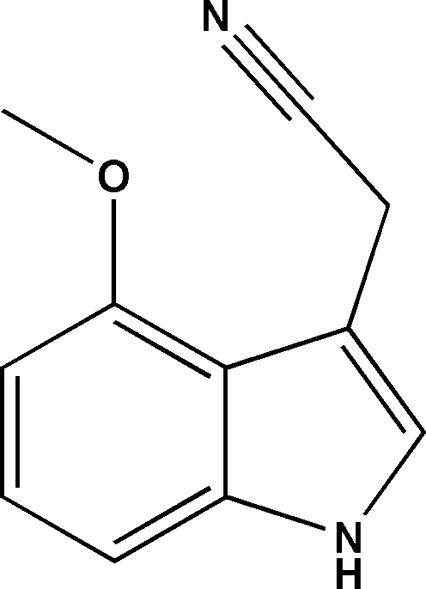


## Experimental

### 

#### Crystal data

C_11_H_10_N_2_O
*M* *_r_* = 186.21
Monoclinic, 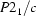

*a* = 8.3182 (17) Å
*b* = 13.062 (3) Å
*c* = 9.3867 (19) Å
β = 101.53 (3)°
*V* = 999.3 (3) Å^3^
*Z* = 4
Mo *K*α radiation
μ = 0.08 mm^−1^
*T* = 293 K
0.26 × 0.24 × 0.15 mm

#### Data collection

Rigaku SCXmini CCD diffractometer
Absorption correction: multi-scan (*CrystalClear*; Rigaku, 2005 ▶) *T*_min_ = 0.979, *T*_max_ = 0.988
9998 measured reflections
2283 independent reflections
1235 reflections with *I* > 2σ(*I*)
*R*_int_ = 0.109

#### Refinement

*R*[*F* ^2^ > 2σ(*F* ^2^)] = 0.088
*wR*(*F* ^2^) = 0.298
*S* = 1.07
2283 reflections
127 parameters
12 restraints
H-atom parameters constrained
Δρ_max_ = 0.38 e Å^−3^
Δρ_min_ = −0.45 e Å^−3^

### 

Data collection: *CrystalClear* (Rigaku, 2005 ▶); cell refinement: *CrystalClear*; data reduction: *CrystalClear*; program(s) used to solve structure: *SHELXS97* (Sheldrick, 2008 ▶); program(s) used to refine structure: *SHELXL97* (Sheldrick, 2008 ▶); molecular graphics: *DIAMOND* (Brandenburg & Putz, 2005 ▶); software used to prepare material for publication: *SHELXL97*.

## Supplementary Material

Crystal structure: contains datablock(s) I, global. DOI: 10.1107/S1600536811053360/hb6547sup1.cif

Structure factors: contains datablock(s) I. DOI: 10.1107/S1600536811053360/hb6547Isup2.hkl

Supplementary material file. DOI: 10.1107/S1600536811053360/hb6547Isup3.cml

Additional supplementary materials:  crystallographic information; 3D view; checkCIF report

## Figures and Tables

**Table 1:** Hydrogen-bond geometry (Å, °)

*D*—H⋯*A*	*D*—H	H⋯*A*	*D*⋯*A*	*D*—H⋯*A*
N1—H1*A*⋯N2^i^	0.86	2.19	3.028 (4)	164

Symmetry code: (i) 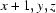.

**Fig. 1.**
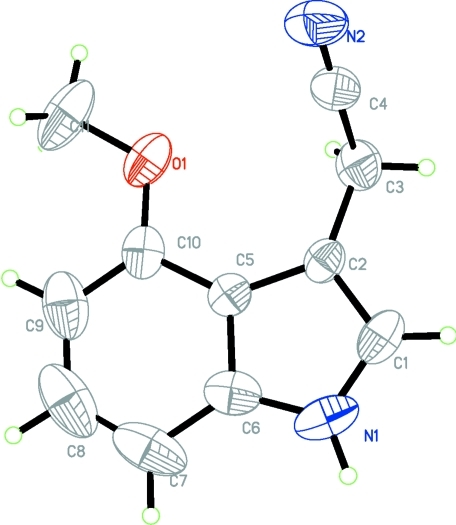
The molecular structure of the title compound with displacement ellipsoids drawn at the 30% probability level.

**Fig. 2.**
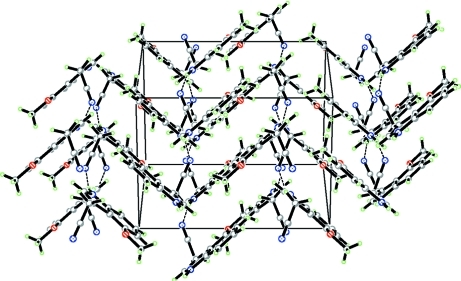
A packing view down the a axis showing hydrogen bonds as dashed lines.

## supplementary crystallographic information

### Experimental

Colourless blocks of the title compound, obtained from a commercial source, were obtained by slow evaporation of a methanol solution.

### Refinement

All H atoms attached to C atoms and O atoms were fixed geometrically and treated as riding with C—H = 0.93 Å (CH), C—H = 0.97 Å (CH_2_), C—H = 0.96 Å (CH_3_)and N—H = 0.86 Å with *U*_iso_(H) = 1.2*U*_eq_(CH, CH_2_ and NH) and *U*_iso_(H) = 1.5*U*_eq_(CH_3_).

### Crystal data

**Table:**

C_11_H_10_N_2_O	*F*(000) = 392
*M**_r_* = 186.21	*D*_x_ = 1.238 Mg m^−^^3^
Monoclinic, *P*2_1_/*c*	Mo *K*α radiation, λ = 0.71073 Å
Hall symbol: -P 2ybc	Cell parameters from 2283 reflections
*a* = 8.3182 (17) Å	θ = 3.1–27.5°
*b* = 13.062 (3) Å	µ = 0.08 mm^−^^1^
*c* = 9.3867 (19) Å	*T* = 293 K
β = 101.53 (3)°	Block, colourless
*V* = 999.3 (3) Å^3^	0.26 × 0.24 × 0.15 mm
*Z* = 4	

### Data collection

**Table:**

Rigaku SCXmini CCD diffractometer	2283 independent reflections
Radiation source: fine-focus sealed tube	1235 reflections with *I* > 2σ(*I*)
graphite	*R*_int_ = 0.109
Detector resolution: 13.6612 pixels mm^-1^	θ_max_ = 27.5°, θ_min_ = 3.1°
CCD_Profile_fitting scans	*h* = −10→10
Absorption correction: multi-scan (*CrystalClear*; Rigaku, 2005)	*k* = −16→16
*T*_min_ = 0.979, *T*_max_ = 0.988	*l* = −12→12
9998 measured reflections	

### Refinement

**Table:**

Refinement on *F*^2^	Primary atom site location: structure-invariant direct methods
Least-squares matrix: full	Secondary atom site location: difference Fourier map
*R*[*F*^2^ > 2σ(*F*^2^)] = 0.088	Hydrogen site location: inferred from neighbouring sites
*wR*(*F*^2^) = 0.298	H-atom parameters constrained
*S* = 1.07	*w* = 1/[σ^2^(*F*_o_^2^) + (0.1429*P*)^2^ + 0.1759*P*] where *P* = (*F*_o_^2^ + 2*F*_c_^2^)/3
2283 reflections	(Δ/σ)_max_ < 0.001
127 parameters	Δρ_max_ = 0.38 e Å^−^^3^
12 restraints	Δρ_min_ = −0.45 e Å^−^^3^

### Special details

**Table:**

Geometry. All e.s.d.'s (except the e.s.d. in the dihedral angle between two l.s. planes) are estimated using the full covariance matrix. The cell e.s.d.'s are taken into account individually in the estimation of e.s.d.'s in distances, angles and torsion angles; correlations between e.s.d.'s in cell parameters are only used when they are defined by crystal symmetry. An approximate (isotropic) treatment of cell e.s.d.'s is used for estimating e.s.d.'s involving l.s. planes.
Refinement. Refinement of *F*^2^ against ALL reflections. The weighted *R*-factor *wR* and goodness of fit *S* are based on *F*^2^, conventional *R*-factors *R* are based on *F*, with *F* set to zero for negative *F*^2^. The threshold expression of *F*^2^ > σ(*F*^2^) is used only for calculating *R*-factors(gt) *etc*. and is not relevant to the choice of reflections for refinement. *R*-factors based on *F*^2^ are statistically about twice as large as those based on *F*, and *R*- factors based on ALL data will be even larger.

### Fractional atomic coordinates and isotropic or equivalent isotropic displacement parameters (Å^2^)

**Table:**

	*x*	*y*	*z*	*U*_iso_*/*U*_eq_	
C5	0.4959 (3)	0.6202 (2)	0.1157 (3)	0.0567 (7)	
C2	0.4374 (4)	0.6934 (2)	0.0033 (3)	0.0610 (8)	
C10	0.4215 (5)	0.5425 (2)	0.1837 (3)	0.0724 (9)	
O1	0.2566 (3)	0.53144 (18)	0.1335 (3)	0.0941 (9)	
N1	0.7052 (4)	0.7163 (3)	0.0794 (4)	0.0927 (11)	
H1A	0.8020	0.7415	0.0864	0.111*	
C6	0.6656 (4)	0.6374 (3)	0.1600 (4)	0.0770 (10)	
N2	0.0524 (4)	0.7726 (3)	0.0570 (4)	0.0978 (11)	
C4	0.1475 (4)	0.7424 (3)	−0.0049 (4)	0.0760 (10)	
C1	0.5681 (5)	0.7495 (3)	−0.0147 (4)	0.0811 (10)	
H1B	0.5653	0.8027	−0.0810	0.097*	
C3	0.2684 (4)	0.7039 (3)	−0.0858 (3)	0.0806 (10)	
H3A	0.2720	0.7501	−0.1661	0.097*	
H3B	0.2323	0.6376	−0.1266	0.097*	
C9	0.5176 (6)	0.4845 (3)	0.2916 (5)	0.1116 (12)	
H9A	0.4708	0.4329	0.3382	0.134*	
C7	0.7644 (6)	0.5791 (3)	0.2705 (4)	0.1120 (12)	
H7A	0.8762	0.5912	0.3007	0.134*	
C8	0.6861 (6)	0.5044 (4)	0.3297 (5)	0.1123 (11)	
H8A	0.7486	0.4634	0.4008	0.135*	
C11	0.1740 (7)	0.4533 (4)	0.1992 (6)	0.141 (2)	
H11A	0.0594	0.4535	0.1553	0.212*	
H11B	0.1872	0.4664	0.3015	0.212*	
H11C	0.2203	0.3877	0.1846	0.212*	

### Atomic displacement parameters (Å^2^)

**Table:**

	*U*^11^	*U*^22^	*U*^33^	*U*^12^	*U*^13^	*U*^23^
C5	0.0627 (17)	0.0571 (15)	0.0523 (15)	−0.0009 (12)	0.0163 (12)	−0.0114 (12)
C2	0.0707 (18)	0.0654 (17)	0.0507 (15)	−0.0105 (13)	0.0213 (13)	−0.0056 (13)
C10	0.099 (2)	0.0579 (17)	0.0641 (19)	0.0040 (16)	0.0265 (18)	−0.0044 (13)
O1	0.107 (2)	0.0782 (16)	0.1058 (19)	−0.0287 (13)	0.0425 (16)	0.0047 (13)
N1	0.0673 (18)	0.104 (2)	0.116 (3)	−0.0194 (16)	0.0396 (18)	−0.041 (2)
C6	0.074 (2)	0.082 (2)	0.077 (2)	0.0092 (17)	0.0204 (17)	−0.0287 (18)
N2	0.0590 (17)	0.125 (3)	0.104 (3)	−0.0091 (17)	0.0054 (17)	−0.009 (2)
C4	0.0611 (19)	0.089 (2)	0.071 (2)	−0.0100 (17)	−0.0037 (16)	0.0014 (17)
C1	0.097 (3)	0.077 (2)	0.082 (2)	−0.0183 (18)	0.048 (2)	−0.0083 (17)
C3	0.086 (2)	0.098 (2)	0.0561 (18)	−0.0022 (18)	0.0085 (16)	0.0000 (16)
C9	0.146 (2)	0.0971 (19)	0.0851 (18)	0.054 (2)	0.0086 (18)	−0.0107 (15)
C7	0.140 (3)	0.102 (2)	0.0871 (18)	0.058 (2)	0.0063 (18)	−0.0196 (15)
C8	0.145 (2)	0.0994 (19)	0.0859 (17)	0.059 (2)	0.0063 (17)	−0.0142 (14)
C11	0.195 (5)	0.090 (3)	0.167 (5)	−0.063 (3)	0.105 (4)	−0.013 (3)

### Geometric parameters (Å, °)

**Table:**

C5—C6	1.407 (4)	C4—C3	1.465 (6)
C5—C10	1.407 (4)	C1—H1B	0.9300
C5—C2	1.435 (4)	C3—H3A	0.9700
C2—C1	1.350 (4)	C3—H3B	0.9700
C2—C3	1.492 (4)	C9—C8	1.400 (7)
C10—O1	1.365 (4)	C9—H9A	0.9300
C10—C9	1.384 (5)	C7—C8	1.353 (7)
O1—C11	1.437 (4)	C7—H7A	0.9300
N1—C6	1.357 (5)	C8—H8A	0.9300
N1—C1	1.366 (5)	C11—H11A	0.9600
N1—H1A	0.8600	C11—H11B	0.9600
C6—C7	1.411 (5)	C11—H11C	0.9600
N2—C4	1.142 (5)		
			
C6—C5—C10	119.2 (3)	C4—C3—H3A	108.7
C6—C5—C2	106.4 (3)	C2—C3—H3A	108.7
C10—C5—C2	134.3 (3)	C4—C3—H3B	108.7
C1—C2—C5	106.9 (3)	C2—C3—H3B	108.7
C1—C2—C3	124.9 (3)	H3A—C3—H3B	107.6
C5—C2—C3	128.1 (3)	C10—C9—C8	119.2 (5)
O1—C10—C9	126.0 (4)	C10—C9—H9A	120.4
O1—C10—C5	115.0 (3)	C8—C9—H9A	120.4
C9—C10—C5	119.0 (4)	C8—C7—C6	115.7 (5)
C10—O1—C11	117.4 (4)	C8—C7—H7A	122.2
C6—N1—C1	109.7 (3)	C6—C7—H7A	122.2
C6—N1—H1A	125.2	C9—C8—C7	124.7 (4)
C1—N1—H1A	125.2	C9—C8—H8A	117.6
N1—C6—C5	107.3 (3)	C7—C8—H8A	117.6
N1—C6—C7	130.4 (4)	O1—C11—H11A	109.5
C5—C6—C7	122.2 (4)	O1—C11—H11B	109.5
N2—C4—C3	179.4 (4)	H11A—C11—H11B	109.5
C2—C1—N1	109.6 (3)	O1—C11—H11C	109.5
C2—C1—H1B	125.2	H11A—C11—H11C	109.5
N1—C1—H1B	125.2	H11B—C11—H11C	109.5
C4—C3—C2	114.1 (2)		

### Hydrogen-bond geometry (Å, °)

**Table:**

*D*—H···*A*	*D*—H	H···*A*	*D*···*A*	*D*—H···*A*
N1—H1A···N2^i^	0.86	2.19	3.028 (4)	164

Symmetry codes: (i) *x*+1, *y*, *z*.
